# Lower Melatonin Secretion in Older Females: Gender Differences Independent of Light Exposure Profiles

**DOI:** 10.2188/jea.JE20140035

**Published:** 2015-01-05

**Authors:** Kenji Obayashi, Keigo Saeki, Nobuhiro Tone, Junko Iwamoto, Kimie Miyata, Yoshito Ikada, Norio Kurumatani

**Affiliations:** 1 Department of Community Health and Epidemiology, Nara Medical University School of Medicine, Nara, Japan; 1 奈良県立医科大学 地域健康医学講座; 2 Center for Academic Industrial and Governmental Relations, Nara Medical University School of Medicine, Nara, Japan; 2 奈良県立医科大学 産学官連携推進センター; 3 Department of Ophthalmology, Nara Medical University School of Medicine, Nara, Japan; 3 天理医療大学 看護学科; 4 Department of Surgery, Nara Medical University School of Medicine, Nara, Japan; 4 奈良県立医科大学 眼科学講座; 5 Department of Nursing, Tenri Health Care University, Nara, Japan; 5 奈良県立医科大学 外科学講座

**Keywords:** melatonin, older people, gender differences, light exposure, 6-sulfatoxymelatonin

## Abstract

**Background:**

Melatonin is associated with a variety of diseases in advanced age, including insomnia, depression, and dementia, and its secretion is influenced by light exposure. Although studies in young and middle-aged subjects have shown that females tend to have higher melatonin levels than males, gender differences in melatonin levels among older people remain unclear.

**Methods:**

To determine the gender differences in melatonin levels among older people in home settings, we conducted a cross-sectional study in 528 older people. We measured overnight urinary 6-sulfatoxymelatonin excretion (UME; an index of melatonin secretion), and ambulatory light intensity.

**Results:**

The mean age of females was 1.8 years younger, and average intensity of daytime light exposure was half that in males (*P* < 0.01). In a univariate comparison, UME was significantly lower in females than in males (*P* < 0.01). A multivariate model using analysis of covariance showed that log-transformed UME remained significantly lower in females after adjustment for potential confounding factors, including age and daytime and nighttime light exposure profiles (males vs. females: 1.90 vs. 1.73 log µg; adjusted mean difference 0.17 log µg [95% confidence interval [CI] 0.02–0.32]; *P* = 0.02). This result indicates that older females have 18.4% (95% CI, 2.2–37.4%) lower UME than older males.

**Conclusions:**

Older females have significantly lower UME than older males, an association which is independent of light exposure profiles in home settings. Our findings may be useful as basic data for further research to investigate gender differences in several diseases associated with melatonin in the elderly.

## Abstract (ja)

**背景:**

メラトニンは不眠症、うつ病、認知症など高齢者に多い疾患との関連が指摘されており、その分泌は光曝露の影響を受ける。メラトニン分泌量は男性に比べて女性に多いという若年あるいは中高年者を対象にした先行研究はあるが、高齢者におけるメラトニン分泌量の性差は十分分かっていない。

**方法:**

高齢者の日常生活下におけるメラトニン分泌量の性差を明らかにするために、我々は528人を対象に横断解析を行った。我々はメラトニン分泌量の指標として夜間尿中メラトニン主要代謝産物排泄量（6-sufatoxymelatonin）を測定し、同時に光曝露量の測定を行った。

**結果:**

平均年齢は女性の方が1.8歳若く、日中光曝露量は男性と比べて女性で半分程度であった（P < 0.01）。単変量分析では、メラトニン分泌量は有意に女性で低下していた（P < 0.01）。共分散分析を用いた多変量分析においても同様に、メラトニン分泌量は女性で低下しており、その性差は年齢、日中夜間の光曝露量を含む潜在的交絡因子と独立していた（調整後平均：男性 1.90 log μg vs. 女性 1.73 log μg; 調整後平均の差：0.17 log μg, 95信頼区間 0.02-0.32; P = 0.02）。この結果は女性高齢者のメラトニン分泌量が男性高齢者と比べて、18.4%（95信頼区間 2.2-37.4%）低下していたということを示している。

**結論:**

女性高齢者のメラトニン分泌量は男性高齢者と比べて有意に低下しており、その性差は日常生活下の光曝露量と独立していた。これらの結果は高齢者におけるメラトニンが関連する疾患の性差を検討する上で、重要な基礎データになるだろう。

## INTRODUCTION

Melatonin is a pineal gland hormone secreted predominantly at night, and serum levels of melatonin are characterized by a peak that occurs in the middle of the night. Nighttime serum melatonin production is accurately reflected by nocturnal urinary 6-sulfatoxymelatonin excretion (UME), which is discharge of the major metabolite of melatonin after rapid metabolism.^1^ Melatonin has been reported to be associated with a variety of diseases in the elderly, including insomnia, depression, and dementia.^2^^–^^7^ Prevalence of these diseases is more frequent in older females than males, and the association of these melatonin-related diseases with females may be partly explained by gender differences in melatonin levels among older people.^8^^–^^10^ Some previous studies in young and middle-aged subjects have reported that females have higher melatonin levels than males.^11^^–^^15^ A previous study has also reported that older females have higher melatonin levels than older males; however, the study had limitations, including single measurement of serum melatonin rather than urinary melatonin, relatively small sample size, and lack of measurement of light exposure.^16^

Physiologically, light exposure is the most effective environmental cue for melatonin secretion in humans. Several studies have reported a positive association between daytime light exposure and nocturnal melatonin levels.^3^^,^^17^ Nighttime light exposure is a powerful suppressor of nocturnal melatonin secretion through the activation of the suprachiasmatic nucleus of the hypothalamus, which contains the master biological clock.^18^ Therefore, adjustment for light exposure profiles is necessary when considering gender differences in melatonin secretion in home settings. A large sample size is also needed to assess gender differences in nocturnal melatonin secretion because of large individual differences in the amount of melatonin secreted.^14^ We aimed to determine gender differences in nocturnal melatonin secretion among older people.

## METHODS

### Participants

A total of 537 older people, ranging from 60 to 90 years, voluntarily enrolled in the “Housing Environments and Health Investigation among Japanese Older People in Nara, Kansai Region: a prospective community-based cohort (HEIJO-KYO) study conducted between late September and early April in 2010, 2011, and 2012. Of these, 528 home-dwelling participants met the inclusion criteria, which required an age ≥60 years and complete UME measurements. All participants provided written informed consent, and the study protocol was approved by the ethics committee of Nara Medical University (No. 301). In this study, we evaluated overnight UME, as an index of nocturnal total melatonin secretion, and ambulatory daytime and nighttime light exposure in 528 older people (247 males and 281 females), most of whom had retired from their jobs.

### Study protocol

Our previously reported study included the protocols for measuring UME and light exposure.^17^ We visited each participant’s home, mostly on weekdays, and collected demographic and medical information during a face-to-face interview administered by trained interviewers using a standardized questionnaire. We measured body weight and height and collected a venous sample. In addition, a 48-h session of environmental light measurement using a set of instruments was started, and all participants were instructed to collect their urine the following night and to maintain a standardized sleep diary logging in-bed time, out-of-bed time, and duration in bed. They were also instructed not to consume any alcohol during the designated monitoring period. Finally, we revisited the participant’s home to retrieve the instruments and collect the urine sample and the sleep diary.

### UME measurement

The urine collection protocol involved discarding the last void at bedtime and collecting each subsequent void until the first morning void. The samples were stored in dark bottles at room temperature, and total volume was measured, after which the samples were stored at −20°C until assay. Urinary 6-sulfatoxymelatonin concentration was measured at a commercial laboratory (SRL, Inc., Tokyo, Japan) using a highly sensitive ELISA kit (RE54031; IBL International, Hamburg, Germany). UME was then calculated as follows: UME (µg) = 6-sulfatoxymelatonin concentration (µg/mL) × total overnight urine volume (mL). UME data were considered missing if alcohol was consumed during the monitoring period or if the urine was not collected in accordance with the protocol. The reproducibility of UME among the initial 192 participants was assessed via additional urine collection approximately 4 months later. Intra-individual coefficient of variation (CV) was calculated using mean values of the first and second measurements and their standard deviation (SD). Inter-individual CV was calculated using mean values of the two measurements of each participant and their SD. The 4 month intra-individual CV was 2.1%, the inter-individual CV was 29.9%, and the intra-class correlation coefficient (ICC) between the two UME levels in the 188 participants (four missing) was 0.66 (95% confidence interval [CI] 0.57–0.73). The ICCs in males and females were 0.72 (95% CI 0.61–0.81) and 0.56 (95% CI 0.41–0.69), respectively.

### Measurements of light exposure

Daytime and nighttime light exposure were measured at 1-min intervals using a wrist light meter (Actiwatch 2; Respironics Inc., Murrysville, PA, USA), worn on the non-dominant wrist, and a portable light meter (LX-28SD; Sato Shouji Inc., Kanagawa, Japan) set in the bedroom, respectively. All participants were instructed not to cover the sensor with their clothing and to tuck their sleeves up using special rubber bands. Values <1 lux during the out-of-bed period were considered artifacts due to clothing covering the sensor and were not included in the analyses.^19^ When duration of missing data exceeded half of the out-of-bed period, the parameters were treated as blank data. The four parameters of daytime and nighttime light exposure were defined as follows:

• the average light intensity during the out-of-bed period (DLavg)
• the total minutes of light ≥1000 lux during the out-of-bed period (DL ≥1000)
• the average light intensity during the in-bed period (NLavg)
• the total minutes of light ≥10 lux during the in-bed period (NL ≥10)

In our previous study,^17^ the day-to-day correlations of DLavg and NLavg between the two days were moderately high (Spearman’s rank correlation coefficients 0.61 and 0.66, respectively).

### Other measurements

Body mass index (BMI) was calculated as weight (kg)/height (m)^2^. Current smoking status, habitual alcohol consumption, past cataract surgery status, and benzodiazepine use were evaluated by a questionnaire. Venous blood samples were analyzed at a commercial laboratory (SRL, Inc.) using a standard clinical chemistry analyzer to determine creatinine level. The estimated glomerular filtration rate (eGFR) was calculated using the Japanese Society of Nephrology-Chronic Kidney Disease Practice Guide formula: eGFR (mL/min/1.73 m^2^) = 194 × (Serum creatinine [mg/dl])^−1.094^ × (Age [years])^−0.287^. The result was multiplied by a correction factor of 0.739 for females. Day length in Nara (latitude 34° N) from sunrise to sunset on measurement days was extracted from the National Astronomical Observatory of Japan website.^20^ Daytime physical activity was also evaluated by the Actiwatch 2 and was defined as the average of all valid physical activity counts per minute during the out-of-bed period.

### Statistical analysis

Normally-distributed variables were expressed as mean ± SD and variables with an asymmetrical distribution as median and interquartile range (IQR). Data on day length, physical activity, sleep parameters, and light exposure parameters were expressed as the mean of measurements over two days. Comparisons between males and females were performed using unpaired *t* tests for continuous data with a normal distribution, the Mann-Whitney test for continuous data with an asymmetrical distribution, and the chi-square test for categorical data. Gender differences in UME were evaluated using analysis of covariance (ANCOVA) with covariates, including age, BMI, smoking and drinking status, self-reported past cataract surgery, benzodiazepine use, eGFR, day length, daytime physical activity, in-bed time, out-of-bed time, duration in bed, and daytime and nighttime light exposure. UME and daytime light exposure parameters were natural log transformed for analysis due to skewed distribution. Because nighttime light parameters and day length were not normally distributed even after natural log transformation, we used categorical analysis of quartile groups for these variables. Statistical analyses were performed using SPSS version 19.0 for Windows (IBM SPSS Inc., Chicago, IL, USA), and a two-sided *P*-value of <0.05 was considered statistically significant.

## RESULTS

Of the 528 participants, 281 (53.3%) were females, with a significantly lower mean age than males (72.0 vs. 73.8 years, *P* < 0.01; Table 1). Mean in-bed time was earlier in males; however, mean out-of-bed time did not differ between males and females. Females were exposed to a shorter day length than males by approximately 25 min. Mean daytime physical activity was significantly higher in females than in males (325.7 vs. 256.1 counts/min, *P* < 0.01). Median UME was significantly lower in females than in males (males vs. females, 7.5 vs. 5.9 µg, *P* < 0.01). Median DLavg and DL ≥1000 were significantly lower in females than in males (262.4 vs. 505.5 lux, and 37.5 vs. 70.8 min, respectively; both *P* < 0.01). Median NLavg and NL ≥10 were marginally but non-significantly lower in females than in males (*P* = 0.19 and *P* = 0.06, respectively).

**Table 1.** Gender comparisons in demographic and clinical parameters

Variables	Males	Females	*P*
	(*n* = 247)	(*n* = 281)	
Age, mean, years	73.8 (6.4)	72.0 (6.5)	<0.01
Body mass index, mean, kg/m^2^	23.3 (2.7)	22.4 (3.3)	<0.01
Current smoker, number	22 (8.9)	1 (0.4)	<0.01
Alcohol consumption (≥30 g/day), number	62 (25.1)	1 (0.4)	<0.01
Self-reported post cataract surgery, number	32 (13.0)	51 (18.1)	0.10
Benzodiazepine use, number	24 (9.7)	40 (14.2)	0.11
eGFR, mean, ml/min/1.73 m^2^	70.0 (13.3)	74.0 (15.5)	<0.01
Day length, median, min	670.0 (613.5–718.0)	645.0 (604.0–697.0)	0.01
Daytime physical activity, mean, count/min	256.1 (95.9)	325.7 (102.7)	<0.01
Sleep parameters			
In-bed time, mean, 24-h clock time	22:18 (01:09)	22:41 (01:05)	<0.01
Out-of-bed time, mean, 24-h clock time	06:47 (01:03)	06:47 (00:50)	0.90
Duration in bed, mean, min	509.2 (80.7)	486.0 (72.3)	<0.01
Melatonin parameters			
UME, median, µg	7.5 (4.3–11.1)	5.9 (3.6–9.0)	<0.01^a^
Log-transformed UME, mean, log µg	1.92 (0.71)	1.75 (0.66)	<0.01
Light exposure parameters, median			
Daytime light			
DLavg, lux	505.5 (227.2–1103.4)	262.4 (148.1–512.3)	<0.01^a^
DL ≥1000, min	70.8 (35.0–133.9)	37.5 (22.5–71.5)	<0.01^a^
Nighttime light			
NLavg, lux	0.9 (0.2–4.0)	0.7 (0.1–3.1)	0.19
NL ≥10, min	9.0 (1.0–27.5)	5.3 (0.5–24.9)	0.06

eGFR, estimated glomerular filtration rate; DLavg, average intensity of daytime light exposure; DL ≥1000, duration of daytime light exposure ≥1000 lux; NLavg, average intensity of nighttime light exposure; NL ≥10, duration of nighttime light exposure ≥10 lux; UME, urinary 6-sulfatoxymelatonin excretion.

Data are reported as means (SD), medians (interquartile range), or number (%).

^a^Compared using unpaired *t*-test after log-transformation of the data.

Gender differences in UME stratified by age (early old age, <75 years; and late old age, ≥75 years) are shown in Figure. Median UME was significantly lower in females than in males in both age groups (males vs. females: 8.5 vs. 6.2 µg, *P* = 0.01 in early old age; 6.8 vs. 5.5 µg, *P* = 0.03 in late old age). Median UME was significantly higher in the early old age group than the late old age group in both males and females (8.5 vs. 6.8, *P* = 0.02; and 6.2 vs. 5.5, *P* = 0.02, respectively).

**Figure.**
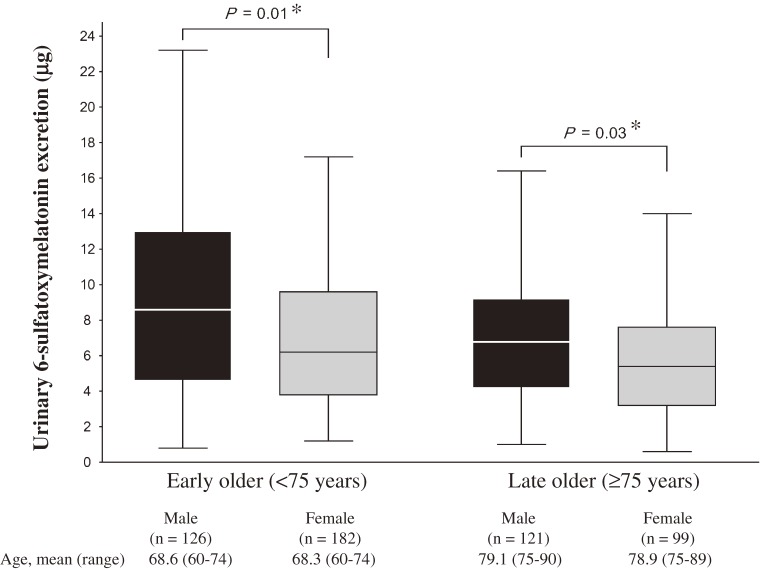
Gender differences in urinary melatonin excretion by age category. Data of urinary 6-sulfatoxymelatonin excretion are presented as medians and 25th and 75th percentiles in males and females (black and grey boxes, respectively), and the lowest value within the 1.5 interquartile range (IQR) of the lower quartile, and the highest value within the 1.5 IQR of the upper quartile (whiskers). *Compared using unpaired *t*-test after log-transformation of urinary 6-sulfatoxymelatonin excretion.

A multivariate comparison of UME using ANCOVA (Table 2) showed that log-transformed UME was significantly lower in females than in males. These gender differences were independent of the covariates, including age, BMI, smoking and drinking status, self-reported past cataract surgery status, benzodiazepine use, eGFR, day length, daytime physical activity, in-bed time, out-of-bed time, duration in bed, and daytime and nighttime light exposure (males vs. females: 1.90 vs. 1.73 log µg; adjusted mean difference 0.17 log µg [95% CI 0.02–0.32]; *P* = 0.02). This adjusted mean difference indicated that older females have, on average, 18.4% (95% CI 2.2–37.4%) lower melatonin levels than older males.

**Table 2.** Gender differences in adjusted mean urinary melatonin excretion using ANCOVA

	Adjusted mean (95% CI)		Adjusted difference (95% CI)	*P*
	
	Male	Female	Male − Female	
Log-transformed UME (log µg)	1.90 (1.81–2.00)	1.73 (1.64–1.83)	0.17 (0.02–0.32)	0.02

ANCOVA, analysis of covariance; CI, confidence interval; DLavg, average intensity of daytime light exposure; NLavg, average intensity of nighttime light exposure; UME, urinary melatonin excretion.

Data are adjusted for age, body mass index, current smoking status, habitual alcohol comsumption, benzodiazepine use, estimated glomerular filtration rate, day length, daytime physical activity, bed-in time, bed-out time, duration in bed, log-transformed DLavg, and NLavg.

## DISCUSSION

Our finding that older females have lower melatonin levels than older males is inconsistent with the findings of a previous study that had limitations in measurement and analysis of melatonin levels. In the previous study,^16^ nocturnal serum melatonin levels were measured only at 2:00 am (clock time) to estimate peak levels. The study found that nocturnal serum melatonin levels were higher in females than in males among an older population; however, the clock time of peak melatonin levels may differ between males and females because of differences in in-bed time.^21^ In addition, light exposure profiles were not taken into account, and a relatively small sample of 73 males and 53 females was included in that previous study. To the best of our knowledge, the present study is the largest study detailing gender differences in endogenous melatonin levels among older people.

The strength of the present study included the collection of extensive information on light exposure profiles as potential confounding factors. Previous studies demonstrated a positive association between daytime light exposure and melatonin levels and a negative association between nighttime light and melatonin levels.^3^^,^^17^^,^^18^ Thus, inconsistent findings for gender differences in melatonin levels between the previous^16^ and the present study may have been caused by differences in light exposure profiles. In the present study, intensity of daytime light in females was half as high as that in males, which is consistent with the findings from previous studies.^22^^,^^23^ In addition, nighttime light levels were marginally but non-significantly lower in females than in males. We observed significant gender differences in melatonin levels after adjustment for light exposure profiles.

The gender differences in melatonin levels that we observed in older people were also inconsistent with those reported in young and middle-aged subjects.^11^^–^^13^ In these studies, nocturnal melatonin levels were measured using nocturnal urinary excretion and multiple nocturnal serum samples; the studies showed that nocturnal melatonin levels were higher in females than males. In contrast, a recent study, including multiple nocturnal salivary samples among the young and middle-aged, reported no significant gender differences in nocturnal melatonin levels,^14^ while other studies have reported that melatonin levels are influenced by menstrual phase and hormonal birth control.^14^^,^^24^ All of the female participants in our study were postmenopausal, and only four were taking sex-hormone-related medication (ie, sex hormone drugs or selective estrogen receptor modulators; data not shown). Our findings may suggest that endogenous melatonin levels are lower in older females because their levels of endogenous sex steroid hormones have decreased, although a previous study in middle-aged females did not detect the association between nocturnal melatonin levels and endogenous sex steroid hormone.^25^ Further research is needed to better understand the association between endogenous sex steroid hormones and melatonin levels in older females.

Among older people, several diseases related to melatonin may be associated with deficiency of endogenous melatonin in females. Melatonin can be transported through the blood-brain barrier owing to its small particle size and lipophilic nature, and it has local beneficial effects on sleep quality, mood, and cognitive function.^2^^,^^4^^–^^7^^,^^26^ In addition, deficiency in endogenous melatonin levels has been observed in patients with insomnia, depression, and dementia.^3^^,^^27^^,^^28^ In our previous study, endogenous melatonin levels were also associated with abnormal circadian blood pressure pattern.^29^ However, an analysis of continuous variables between endogenous melatonin levels and these diseases has not yet been conducted; therefore, further research is required to determine any clinical implications of the difference noted in the present study in endogenous melatonin levels between older females and older males.

The present study includes some limitations. First, we collected a single overnight urine sample rather than multiple samples, possibly leading to misclassification of the melatonin status of some participants. However, the moderately high ICC in males and females that we observed between two UME levels among initial participants suggested that serious misclassification was infrequent. Second, the present study was conducted in the colder months from late September to early April. We were therefore unable to appropriately assess the effect of seasonal variations on melatonin levels, although the final model of gender differences in UME included day length as a confounding factor, which is the most important seasonal cue for melatonin secretion. However, the model did not include any weather information. Finally, we employed nonrandom sampling because participants were recruited with the cooperation of local resident associations and senior citizens clubs, potentially leading to selection bias. However, the generalizability of our findings may be acceptable given that some basic data (eg, BMI and eGFR) and the proportion of antihypertensive medication use (45.1%) in our participants were consistent with those in the random sample of people aged over 60 years in the National Health and Nutrition Survey Japan in 2010.^30^

In conclusion, we found that older females have significantly lower melatonin levels than older males, a relationship which is independent of potential confounding factors, including age and daytime and nighttime light exposure profiles in home settings. Our findings may be useful as basic data for further investigations of gender differences in risk of several diseases associated with melatonin in elderly populations.

## ONLINE ONLY MATERIAL

Abstract in Japanese.
