# Supplementary material for: Lower Melatonin Secretion in Older Females: Gender Differences Independent of Light Exposure Profiles
Source: J Epidemiol. 2015 Jan 5;25(1):38–43. doi: 10.2188/jea.JE20140035 (PMC4275436; doi:10.2188/jea.JE20140035)
Supplement: Abstract in Japanese. [file je-25-038-s001.pdf]

女性高齢者における低いメラトニン分泌量： 光曝露情報と独立した性差

大林賢史[1]、佐伯圭吾[1]、刀根庸浩[2]、岩本淳子[3]、宮田季美恵[4]、　筱義人[5]、　車谷典男[1]

[1]奈良県立医科大学 地域健康医学講座、[2]奈良県立医科大学 産学官連携推進センター
[3]天理医療大学 看護学科、[4]奈良県立医科大学 眼科学講座、[5]奈良県立医科大学 外科学講座、

背景
メラトニンは不眠症、うつ病、認知症など高齢者に多い疾患との関連が指摘されており、その分泌は光曝露の影響を受ける。メラトニン分泌量は男性に比べて女性に多いという若年あるいは中高年者を対象にした先行研究はあるが、高齢者におけるメラトニン分泌量の性差は十分分かっていない。

方法
高齢者の日常生活下におけるメラトニン分泌量の性差を明らかにするために、我々は 528 人を対象に横断解析を行った。我々はメラトニン分泌量の指標として夜間尿中メラトニン主要代謝産物排泄量（6-sufatoxymelatonin）を測定し、同時に光曝露量の測定を行った。

結果
平均年齢は女性の方が 1.8 歳若く、日中光曝露量は男性と比べて女性で半分程度であった（$P < 0.01$）。単変量分析では、メラトニン分泌量は有意に女性で低下していた（$P < 0.01$）。共分散分析を用いた多変量分析においても同様に、メラトニン分泌量は女性で低下しており、その性差は年齢、日中夜間の光曝露量を含む潜在的交絡因子と独立していた（調整後平均 : 男性 1.90 log μg vs. 女性 1.73 log μg; 調整後平均の差：0.17 log μg, 95 信頼区間 0.02-0.32; $P = 0.02$）。この結果は女性高齢者のメラトニン分泌量が男性高齢者と比べて、18.4%（95 信頼区間 2.2-37.4%）低下していたということを示している。

結論
女性高齢者のメラトニン分泌量は男性高齢者と比べて有意に低下しており、その性差は日常生活下の光曝露量と独立していた。これらの結果は高齢者におけるメラトニンが関連する疾患の性差を検討する上で、重要な基礎データになるだろう。

キーワード
メラトニン、高齢者、性差、光曝露、6-sulfatoxymelatonin
